# Daily Movement Matters: Post-Exercise Hypotension in Peripheral Arterial Disease—A Quasi-Experimental Pilot Study

**DOI:** 10.3390/jfmk10040426

**Published:** 2025-11-03

**Authors:** Saúl Peñín-Grandes, Susana López-Ortiz, Montserrat de la Fuente Gómez, Mª Lourdes del Río-Solá, Sergio Maroto-Izquierdo, Alejandro Santos-Lozano, Juan Martín-Hernández, José Pinto-Fraga

**Affiliations:** 1 i+HeALTH Strategic Research Group, Department of Health Sciences, Miguel de Cervantes European University (UEMC), 47012 Valladolid, Spain; spenin@uemc.es (S.P.-G.); smaroto@uemc.es (S.M.-I.); jmartinh@uemc.es (J.M.-H.); fjpinto@uemc.es (J.P.-F.); 2 Rehabilitation Department, University Hospital of Valladolid, 47003 Valladolid, Spain; mmfuente@saludcastillayleon.es; 3 Vascular Surgery Department, University Hospital of Valladolid, 47003 Valladolid, Spain; mlrio@uemc.es; 4 Physical Activity and Health Research Group (“PaHerg”), Research Institute of Hospital “12 de Octubre” (“imas12”), 28041 Madrid, Spain

**Keywords:** cardiovascular disease, high blood pressure, exercise, circuit-based training, acute exercise

## Abstract

**Background**: Aerobic and resistance training have acute effects on blood pressure (BP) in peripheral arterial disease (PAD). However, the combined effect of both exercises in a single session is still unknown. The aim of this study was to analyze the effects of a single exercise session combining walking and circuit-based training on BP in patients with PAD. **Methods**: Participants with PAD (n = 13; 65.0 ± 10.2 years; 76.9% male) underwent a supervised exercise therapy (SET) intervention (312 sessions, 24 sessions/patient) that included 15–30 min of walking, followed by 15 min circuit-based training. Clinic systolic (SBP) and diastolic (DBP) were recorded 5 min before and after each exercise session. Longitudinal changes were analyzed using repeated-measures analysis of variance (ANOVA) and categorical changes in blood pressure levels were evaluated with chi-square tests. **Results**: After each exercise session, clinic SBP decreased 4.87 mmHg (*p* < 0.001) and clinic DBP decreased 2.11 mmHg (*p* < 0.001). Furthermore, there were no differences between the initial stage of training (1–10 sessions) and late (14–24 sessions) for each time that SBD or DBP were measured. **Conclusions**: After an acute exercise session, both clinical SBP and DBP decreased in patients with PAD compared to pre-exercise values. However, no additional reductions in clinical BP were observed when comparing early (sessions 1–10) and late (sessions 14–24) stages of the full SET intervention.

## 1. Introduction

People with peripheral arterial disease (PAD)—a condition defined by the narrowing and obstruction of the antegrade flow of major systemic arteries, particularly in the lower limbs—have a high prevalence of multiple comorbidities, such as hypertension, diabetes, or hyperlipidemia that enhances the risk of major adverse events [1,2]. Hypertension, the most prevalent one [1,2], is a major cardiovascular risk factor [3], and has a significant impact on global public health in these individuals [4,5]. There is growing evidence that systolic blood pressure (BP) is an important predictor of cardiovascular events, emphasizing the importance of tight BP control in reducing cardiovascular morbidity and mortality. Therefore, clinical interventions should focus on the reduction in this important comorbidity [6].

Supervised exercise therapy (SET) is a well-established non-pharmacological treatment for patients with PAD [7], and has both an acute and chronic positive effect on BP in this patient group [8]. Understanding the acute effects of exercise has emerged as an important aspect of health, as it is well known that there is a window of vulnerability and opportunity following exercise. An acute response observed after exercise is hypotension [9], a phenomenon characterized by a decrease in systolic (SBP) and/or diastolic BP (DBP) following a single bout of exercise compared to resting baseline values. This transient drop in BP, known as post-exercise hypotension, has gained attention as a meaningful clinical marker due to its potential to predict long-term BP adaptations and its relevance in populations with elevated cardiovascular risk [10]. Aerobic exercise (e.g., walking and arm-cranking) and resistance training are strongly recommended in this population and have been shown to lead to post-exercise hypotension in patients with PAD [11,12,13,14,15,16]. In terms of aerobic exercise, intermittent walking exercise protocols consisted of 10 to 15 bouts of 2 min treadmill walking, each separated by 2 min rest intervals [14,15]. In addition, arm-crank exercise consisted of 15 bouts of 2 min, each interspersed with 2 min passive rest intervals [16]. Conversely, resistance training protocols included 6 to 8 exercises performed in 3 sets, with repetitions ranging from 8 to 12 per set with an intensity prescribed either at ~60% of one-repetition maximum (1RM) or based on a perceived exertion of 11 to 13 on the 15-point Borg scale [11,12].

Circuit-based training (i.e., a combination of different resistance exercises performed with short rest periods) has emerged as an effective method to elicit both cardiovascular and neuromuscular stimuli while significantly saving time [17], and it appears to be effective in patients with PAD to improve key functional variables [18]. However, the effects of circuit-based training on the acute clinic BP responses in PAD patients remain unclear. Therefore, this study aimed to analyze the acute effects of each training session of walking and circuit-based training on post-exercise clinic BP in patients with PAD. It was hypothesized that a single combined session of walking and circuit-based training would acutely reduce post-exercise clinic systolic and diastolic BP in patients with PAD, and that regular participation in such sessions would lead to additional long-term improvements in BP.

## 2. Materials and Methods

### 2.1. Study Design

**Figure 1:**
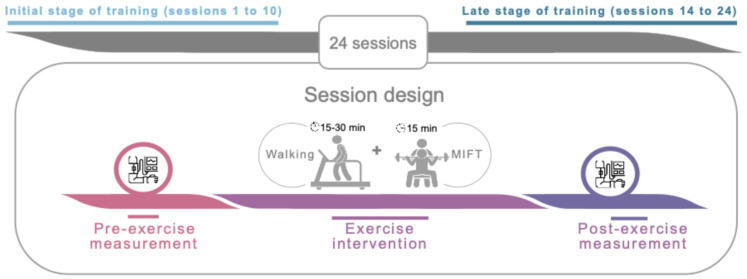
Overview of study design and chronology. Abbreviations: MIFT, moderate-intensity functional training.

We conducted a quasi-experimental pilot study. The study was prepared following the TREND Statement Checklist (see Supplementary Files) All participants underwent a SET intervention (24 sessions) that included 15–30 min of walking, followed by a 15 min circuit-based training. Participants performed five training sessions every two weeks (i.e., week 1: Monday, Wednesday, and Friday; week 2: Tuesday, and Thursday). SBP and DBP were recorded in a seated position 5 min before and 5 min after each training session (see Figure 1).

### 2.2. Participants

Thirteen patients with PAD (see Table 1) signed an informed consent form for voluntary participation in the study at the Department of Vascular Surgery of the University Hospital of Valladolid (Valladolid, Spain). The inclusion criteria were (i) patients with PAD diagnosis in stage IIa or IIb according to the Leriche–Fontaine classification and (ii) age ≥ 18 years old. The exclusion criteria were (i) patients with a major surgical procedure within the last year, (ii) hospitalized patients, (iii) wheelchair-dependent patients and (iv) patients with a history of dementia or psychosocial problems. The study was approved by the University Hospital of Valladolid Ethics Committee (CASVE-NM-19-384) on 21 February 2019. All procedures were conducted in accordance with the 1964 Declaration of Helsinki and its subsequent amendments.

### 2.3. Intervention

All training sessions (312 sessions, 24 sessions/patient) were conducted between 13:00 h and 15:00 h. All sessions began with a standardized 5 min warm-up, consisting of joint mobility (i.e., upper neck, shoulder, thoracic spine, hip, and ankle mobility) and low intensity weight-bearing exercises (i.e., various steps in the frontal, sagittal, and transverse planes). Patients then engaged in up to 15 min of intermittent walking training on a treadmill (F2W DUAL, BH Fitness^®^, Madrid, Spain). Exercise volume was incrementally increased by five min every two weeks, reaching 30 min by the seventh week. Exercise intensity was individually adjusted to induce moderate claudication pain for three to five min. Participants were instructed to stop walking immediately upon experiencing pain and to rest by standing on the mat until the pain subsided. To adjust the intensity in each session, the treadmill speed was first increased to a maximum of six km·h^−1^, followed by increasing the incline in 1% increments.

Five min after intermittent walking, patients performed the circuit-based training for 15 min. During this time, patients were instructed to complete as many repetitions/rounds as possible (AMRAP) in a circuit comprising six functional exercises: (i) kettlebell step-up, (ii) bands standing chest press, (iii) Swiss ball kettlebell squat, (iv) bands standing rowing, (v) farmer walk, and (vi) kettlebell deadlift. Participants performed ten repetitions of each exercise with minimal rest between exercises, maintaining a rating of perceived exertion (RPE) between five and seven out of ten, thus adapting the AMRAP protocol to moderate intensity for safety. They were allowed to determine the length of the rest periods between exercises themselves. Heart rate was monitored during training sessions for safety reasons only.

### 2.4. Outcomes

#### Primary Outcomes

Clinic SBP and DBP (Welch Allyn Spot LXi Vital Signs Monitor, Skaneateles Falls, New York, NY, USA) were measured five min before (pre-SBP and pre-DBP) and after (post-SBP and post-DBP) each training session, following a 5 min rest period in a seated position. This standardized rest period ensures that the measured values reflect stable hemodynamic conditions and that the influence of temporary physiological fluctuations is minimized. The cuff was adjusted to the circumference of the right arm, 2–3 cm above the cubital fossa at heart level. Measurements were performed in triplicate with a 1 min interval between recordings, and the mean value of the three measurements was used for analyses. All assessments were conducted by trained personnel under standardized environmental conditions [e.g., quiet room, constant temperature (20 ± 1 °C) and low light], and participants were instructed to abstain from caffeine, smoking and vigorous physical activity for at least two hours before each session.

### 2.5. Statistical Analyses

Statistical analyses were performed using SPSS 26.0 (SPSS for Windows, Rel. 26.0.0. SPSS, Chicago, IL, USA). All data were expressed as mean ± standard deviation (SD). Data from 24 sessions were included, those completed by all the participants. The normality of distribution assumption was checked by the Shapiro–Wilk test. Differences in SBP and DBP values pre–post session were tested by Student *t* test repeated measures, or the nonparametric alternative, the Wilcoxon test. Repeated-measures ANOVA was used to study longitudinal changes in clinic SPB and DPB due to the interventions. Furthermore, effect sizes were calculated. For continuous variables, the Cohen’s *d* was used, interpreted as small (0.2 ≤ *d* < 0.5), medium (0.5 ≤ *d* < 0.8) or large (*d* ≥ 0.8) effects; for categorical changes [19], Cramér’s *V* was assessed, interpreted as small (0.1 ≤ *V* < 0.3), moderate (0.3 ≤ *V* < 0.5) or large (*V* > 0.5) [20]. Finally, the clinic SBP and DBP variables were categorized into 4 levels (Optimal, Normal, High normal, and Grade 1 Hypertension) following the European Guidelines [21]. Optimal blood pressure is below 120/80 mmHg; normal blood pressure is 120–129 mmHg SBP and 80–84 mmHg DBP; high normal is 130–139 mmHg SBP and 85–89 mmHg DBP; and grade 1 hypertension is >140 mmHg SBP and >90 mmHg DBP [21]. The changes between levels for each participant were evaluated using the chi-square test. Statistical significance was defined as *p* ≤ 0.05.

## 3. Results

Clinical characteristics of the 13 participants enrolled in the study are presented in Table 1. The mean age of participants was 65 ± 10.2 years with 76.9% male. More than half of the participants had stage IIa of the Leriche–Fontaine classification.

**Figure 2:**
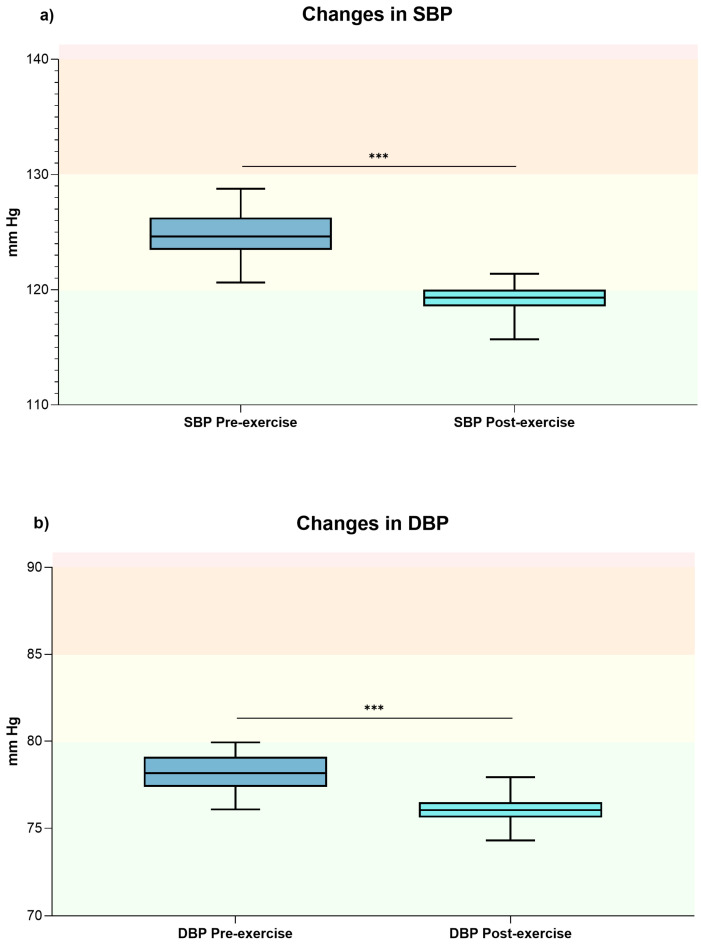
The box plot shows the general changes in clinic SBP (panel (**a**)) and DBP (panel (**b**)) values before and after exercise sessions. *** Indicates *p*-value < 0.001. Optimal blood pressure (green); normal blood pressure (yellow); high normal (orange); and grade 1 hypertension (red) [21]. Abbreviations: DBP, diastolic blood pressure; mmHg, millimeters of mercury; SBP, systolic blood pressure.

After a single exercise session both clinic SBP (pre-SBP 124.17 ± 9.36 mmHg vs. post-SBP 119.30 ± 8.28 mmHg; *p* < 0.001; *d* = 2.3) and DBP (pre-DBP 78.18 ± 6.00 mmHg vs. post-DBP 76.05 ± 5.9 mmHg; *p* < 0.001; *d* = 1.71) showed a significant reduction, both indicating a large effect size (see Figure 2, panels a and b).

More specifically, significant differences were found in the participants with PAD between pre-SBP and post-SBP values in all training sessions except in sessions 2 and 4 (see Supplementary Table S1). Also, significant differences were found between pre-DBP and post-DBP results in all training sessions except in sessions 2, 3, 4, 6, 7, 8, 10, 19, 20 and 24 (see Supplementary Table S1).

Furthermore, there were no significant differences between sessions 1 to 10 (i.e., first sessions) or between sessions 14 to 24 (i.e., last sessions) for each time that SBD or DBP were measured. Thus, measurements 1–10 (initial stage of training) and 14–24 (late stage of training) were pooled for comparison. There were no significant time interactions in SBP [(pre-SBP-initial stage of training: 125.54 ± 13.89 mmHg vs. pre-SBP-late stage of training: 124.17 ± 9.36 mmHg; *p* = 0.074; *d* = 0.16) and (post-SBP-initial stage of training: 119.12 ± 11.87 mmHg vs. post-SBP-late stage of training: 119.30 ± 8.28 mmHg; *p* = 0.776; *d* = −0.02)] and DBP at any evaluation point [(pre-DBP-initial stage of training: 78.18 ± 7.90 mmHg vs. pre-DBP-late stage of training: 77.95 ± 5.07 mmHg; *p* = 0.598; *d* = 0.05) and (post-DBP-initial stage of training: 76.26 ± 7.60 mmHg vs. post-DBP-late stage of training: 75.84 ± 5.23 mmHg; *p* = 0.313; *d* = 0.09)].

**Figure 3:**
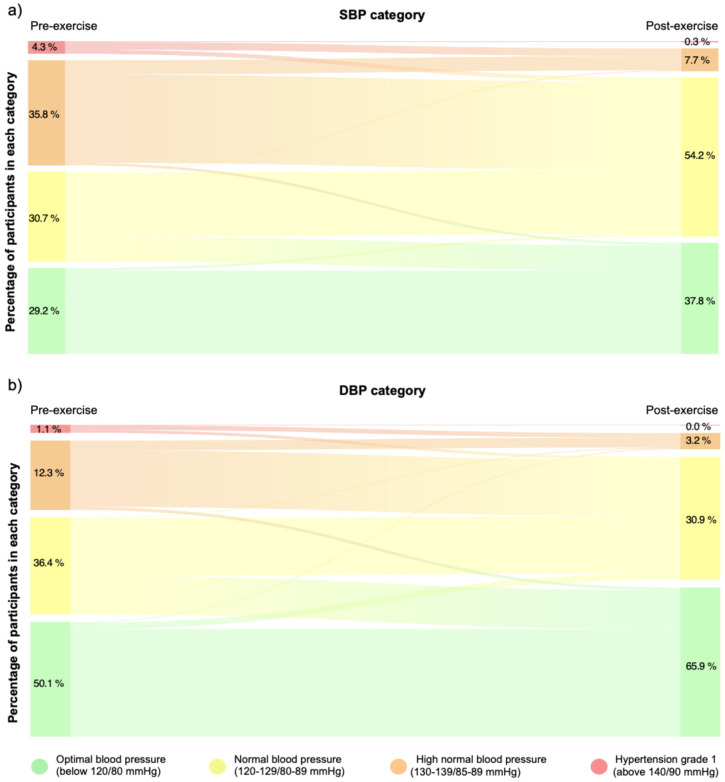
The Sankey diagram shows the percentage of participants who change in clinic SBP (panel (**a**)) and DBP (panel (**b**)) categories before and after exercise sessions. Changes in continuity between bands represent movements of patients from one category to another. Optimal blood pressure (green); normal blood pressure (yellow); high normal (orange); and grade 1 hypertension (red) [21].

Finally, training sessions led to significant changes in clinic SBP (*p* < 0.0001; *V* = 0.38) and DBP (*p* = 0.024; *V* = 0.29) categories when comparing pre- and post-training values in the patients with PAD, indicating moderate effect sizes for both variables (see Figure 3, panels a and b). In addition, 30% of the records move from the high-normal to the optimal category, while 9% decreased from the normal to the optimal category for SBP. Regarding DBP decreases, 9% decreased from the high normal to the normal category, while 18% decreased from the normal to the optimal category.

## 4. Discussion

Our results showed that following an acute exercise session, patients experienced a decrease in both clinic SBP and DBP compared to pre-exercise values, predictably leading to a reduction in participants’ overload and potential cardiovascular risk due to category changes. This immediate hypotensive response is consistent with the concept of post-exercise hypotension, a well-documented and clinically significant reduction in cardiovascular stress, particularly in patients at increased cardiovascular risk [22,23]. Conversely, completing a full exercise program (SET of 24 sessions) did not result in a reduction in their clinical BP levels. This may suggest that while each session produces positive hemodynamic responses, sustained long-term changes may require either more frequent exercise or interventions tailored to individual physiological variability.

For clinical relevance, post-exercise hypotension should involve a significant reduction in BP (>4 mmHg for SBP and >2 mmHg for DBP) and should remain for a prolonged period following exercise [24,25]. The mean magnitude of acute clinic BP reduction observed in this study was significant for SBP (−4.87 ± 5.18 mmHg) and DBP (−2.11 ± 3.15 mmHg). These results are in line with previous studies of patients with symptomatic PAD who performed walking-based SET interventions (systolic greatest net effect: −13 ± 2 mmHg; diastolic greatest net effect: −5 ± 2 mmHg) [14], and resistance exercise training (systolic greatest net effect: −14 ± 5 mmHg; diastolic greatest net effect: −6 ± 5 mmHg) [12]. Although the effect of post-exercise hypotension was demonstrated 5 min after the end of an acute exercise session, studies involving walking and resistance training interventions in patients with PAD have shown a maximal reduction in BP occurring between 10 and 45 min that lasts for up to an hour or even longer after the training session ends [12,14,26]. It is possible that our measurement time point may have missed the maximum hypotensive window, possibly underestimating the full extent of the response. The similarity of the promising results in comparison with other studies involving a different sample size, training protocols (i.e., two bouts of 15 min of intermittent walking exercise or three sets of 12, 10 and 8 repetitions of 6 resistance exercises with a perceived exertion of 11–13 on the 15-grade Borg scale) or assessment time points (i.e., ambulatory BP or 15, 30, 45 and 60 min after exercise session) support the acute post-exercise hypotension effect of exercise in patients with PAD [12,14,26]. This consistency reinforces the external validity of our findings, despite differences in methodology.

The post-exercise hypotension response following concurrent training, which consists of walking and MIFT in patients with PAD is a transient phenomenon. It does not persists for up to 24 h after exercise when patients return to their normal daily activities [27]. However, studies recording ambulatory BP have shown that on days when individuals with higher blood pressure engage in exercise, their blood pressure readings are lower [28]. This finding is significant as it indicates that patients’ BP remains in a lower category for several hours after exercise concludes, serving as a more robust predictor of cardiovascular disease and mortality risk [29]. Thus, exercise frequency (i.e., the number of exercise sessions completed per week) is crucial in determining its effect on post-exercise hypotension. From a clinical perspective, this emphasizes the importance of encouraging patients with PAD to exercise daily or near-daily, not only for functional improvements but also for acute cardiovascular modulation. Therefore, patients with PAD are encouraged to participate regularly in exercise, targeting an increased frequency, as it represents a viable alternative to pharmacological interventions for managing elevated BP in this population.

Based on these considerations, this study could contribute in practice to the effective use of intermittent walking and circuit-based training in clinical and community settings for the acute reduction in BP in patients with PAD. These forms of exercise offer practical, time-saving options that can complement pharmacologic treatments, support cardiovascular health, and potentially improve patient adherence to rehabilitation programs. Importantly, circuit-based protocols also offer psychological and motivational benefits, such as more variety, less perceived effort and more fun, which could further encourage long-term participation [30].

The main limitation of this study was the lack of a control group and the lack of further measurements within min, hours or an ambulatory basis after the end of the training sessions. Furthermore, the relatively small sample size could limit the generalizability of our results and reduce the statistical power to detect more subtle inter-individual differences. However, some strengths should be emphasized. In particular, the intervention and outcomes were systematically monitored by medical professionals and sports scientists, ensuring the safety of the participants during the training sessions. To address these limitations and extend current knowledge, future studies should include control groups and perform ambulatory BP monitoring to better assess the duration and clinical significance of hypotension after exercise. In addition, the effect of different exercise modalities, intensities and frequencies on BP should be investigated to optimize the exercise prescription for patients with PAD. Furthermore, biological sex should be considered as a potential factor influencing cardiovascular responses to exercise, as men and women may exhibit different adaptations that affect the magnitude and duration of post-exercise hypotension. These studies appear promising and hold the potential for significant short-term improvements in participants’ BP and also provide the opportunity to assess the lasting effects of these improvements in the short-, medium- and long-term.

## 5. Conclusions

The present study suggests that a single session of intermittent walking and circuit-based training induced post-exercise hypotension due to a reduction in both clinic SBP and DBP in patients with PAD. No additional reductions in clinical BP were observed when comparing early (sessions 1–10) and late (sessions 14–24) stages of the full SET intervention.

## Figures and Tables

**Table 1:** Baseline characteristics of the participants included in the study.

	n = 13
Gender (n men; %)	10 (76.9)
Age (years)	65.0 ± 10.2
Height (cm)	168.0 ± 8.0
Weight (kg)	72.8 ± 7.9
BMI (kg·m^−2^)	21.6 ± 1.7
Smokers (n; %)	3 (23.1)
**Ankle-Brachial Index**	
Right	0.68 ± 0.28
Left	0.58 ± 0.29
**PAD severity (Leriche–Fontaine classification)**	
IIa (%)	53.84
IIb (%)	46.15
**Surgical interventions**	
Bypass lower extremity (%)	16.67
Stent lower extremity (%)	22.22
Surgical thrombectomy (%)	11.11
**Medical treatment**	
Antiplatelet (%)	84.61
Antihypertensive therapy (%)	76.92

Values are expressed as mean ± SD. Abbreviations: BMI, body mass index; PAD, peripheral arterial disease.

## Data Availability

The data underlying this article will be shared on reasonable request to the corresponding or first author.

## Supplementary Materials

The following supporting information can be downloaded at: https://www.mdpi.com/article/10.3390/jfmk10040426/s1. Table S1. Differences in systolic and diastolic blood pressure before and after exercise in all training sessions. Ref. [31] is cited in Supplementary Materials file.

## Abbreviations

The following abbreviations are used in this manuscript:

AMRAP	As Many Repetitions As Possible
BP	Blood pressure
DBP	Diastolic blood pressure
MIFT	Moderate-intensity functional training
PAD	Peripheral arterial disease
SBP	Systolic blood pressure
SET	Supervised exercise therapy
