# Supplementary material for: Daily Movement Matters: Post-Exercise Hypotension in Peripheral Arterial Disease—A Quasi-Experimental Pilot Study
Source: J Funct Morphol Kinesiol. 2025 Nov 3;10(4):426. doi: 10.3390/jfmk10040426 (PMC12641763; doi:10.3390/jfmk10040426)
Supplement: Supplementary file 1 [file jfmk-10-00426-s001.zip › Supplementary Table S1.pdf]

Supplementary Table S1. Differences in systolic and diastolic blood pressure before and after exercise in all training sessions.

Session	Systolic Blood Pressure			Diastolic Blood Pressure		
	Pre-exercise (mm Hg)	Post-exercise (mm Hg)	Paired t-test	Pre-exercise (mm Hg)	Post-exercise (mm Hg)	Paired t-test
1	127.23 ± 15.91	121.31 ± 12.18	**0.003**	79.31 ± 7.15	75.85 ± 7.02	**0.036**
2	126.46 ± 12.97	121.38 ± 7.65	0.066	78.69 ± 5.78	77.92 ± 5.91	0.468
3	124.00 ± 14.31	117.15 ± 10.21	**0.034**	79.54 ± 8.50	77.15 ± 6.77	0.081
4	126.15 ± 15.35	120.77 ± 9.88	0.059	77.62 ± 8.80	75.85 ± 8.29	0.168
5	126.54 ± 9.63	118.85 ± 12.65	**0.001**	78.92 ± 7.73	75.85 ± 7.99	**0.002**
6	126.31 ± 12.18	119.85 ± 10.79	**0.001**	77.92 ± 7.09	76.54 ± 7.57	0.148
7	128.77 ± 12.26	119.62 ± 11.46	**<0.001**	76.92 ± 8.19	76.38 ± 5.56	0.719
8	124.92 ± 15.46	120.23 ± 15.16	**0.021**	78.31 ± 9.65	76.85 ± 8.75	0.086
9	124.38 ± 16.78	115.69 ± 15.59	**0.000**	77.54 ± 9.34	74.31 ± 11.31	**0.017**
10	120.62 ± 15.90	116.31 ± 13.59	**0.001**	77.08 ± 8.44	75.92 ± 7.50	0.182
11	122.00 ± 12.80	117.54 ± 10.88	**<0.001**	78.85 ± 6.94	75.38 ± 6.75	**<0.001**
12	125.38 ± 11.30	118.85 ± 10.44	**<0.001**	79.92 ± 5.11	76.85 ± 5.97	**<0.001**
13	125.08 ± 11.06	118.46 ± 9.32	**<0.001**	78.69 ± 6.85	76.38 ± 5.88	**0.001**
14	123.38 ± 10.91	118.85 ± 10.50	**<0.001**	77.54 ± 7.41	75.54 ± 6.46	**0.015**
15	124.38 ± 10.77	119.85 ± 9.22	**0.002**	79.31 ± 5.75	77.31 ± 4.35	**0.025**
16	127.00 ± 7.69	120.08 ± 7.08	**<0.001**	79.15 ± 4.02	76.15 ± 4.60	**<0.001**
17	125.92 ± 8.24	120.08 ± 6.68	**<0.001**	79.15 ± 4.51	76.15 ± 4.95	**<0.001**
18	124.85 ± 8.66	119.69 ± 8.45	**<0.001**	77.31 ± 4.55	74.77 ± 5.70	**0.026**
19	123.69 ± 10.44	119.00 ± 8.30	**0.001**	77.31 ± 5.60	75.92 ± 5.07	0.056
20	122.54 ± 9.28	116.69 ± 8.10	**<0.001**	77.69 ± 5.78	75.92 ± 5.58	0.128
21	123.08 ± 10.63	119.31 ± 9.25	**0.021**	78.54 ± 5.29	76.15 ± 6.04	**0.013**
22	124.08 ± 9.46	119.77 ± 8.35	**0.005**	77.00 ± 5.00	74.92 ± 5.09	**0.005**
23	124.08 ± 9.51	119.31 ± 9.38	**<0.001**	78.00 ± 5.61	76.15 ± 5.70	**0.030**
24	122.08 ± 10.66	119.23 ± 9.93	**0.001**	76.08 ± 5.02	74.92 ± 6.29	0.091
**Mean**	124.71 ± 1.91	119.08 ± 1.48	**<0.001**	78.18 ± 0.99	76.05 ± 0.84	**<0.001**
**Q1**	123.61	118.75		77.48	75.77	

**Q3**	126.19	119.91	78.98	76.42
**Median**	124.62	119.31	78.16	76.04

Values are expressed as mean ± SD.
